# High Catalytic Efficiency of Nanostructured β-CoMoO_4_ in the Reduction of the *Ortho*-, *Meta*- and *Para*-Nitrophenol Isomers

**DOI:** 10.3390/molecules23020364

**Published:** 2018-02-09

**Authors:** Fahd Al-Wadaani, Ahmed Omer, Mostafa Abboudi, Hicham Oudghiri Hassani, Souad Rakass, Mouslim Messali, Mohammed Benaissa

**Affiliations:** 1 Chemistry Department, College of Science, Taibah University, Almadinah 30002, Saudi Arabia; fwadaani@taibahu.edu.sa (F.A.-W.); ahmed.hussein.omer@gmail.com (A.O.); abboudi14@hotmail.com (M.A.); rakass_souad@yahoo.fr (S.R.); aboutasnim@yahoo.fr (M.M.); 2 Département Sciences de la Nature, Cégep de Drummondville, 960 Rue Saint-Georges, Drummondville, QC J2C 6A2, Canada; 3 LMPHE, Département de Physique, Faculté des Sciences, Université Mohammed V, B.P. 1014 RP, Rabat 10000, Morocco; benaissa.um5@gmail.com

**Keywords:** nanostructures, β-CoMoO_4_, nitrophenol reduction, nanoparticles

## Abstract

Nanostructured β-CoMoO_4_ catalysts have been prepared via the thermal decomposition of an oxalate precursor. The catalyst was characterized by infrared spectroscopy (FTIR), X-ray diffraction (XRD), Brunauer-Emmett-Teller method (BET), energy dispersive X-ray spectroscopy (EDX), and transmission electron microscopy (TEM). The efficiency of these nanoparticles in the reduction of *ortho*- and *meta*-nitrophenol isomers (2-NP, 3-NP, and 4-NP) to their corresponding aminophenols was tested using UV-visible spectroscopy measurements. It was found that, with a β-CoMoO_4_ catalyst, NaBH_4_ reduces 3-NP instantaneously, whilst the reduction of 2-NP and 4-NP is slower at 8 min. This difference is thought to arise from the lower acidity of 3-NP, where the negative charge of the phenolate could not be delocalized onto the oxygen atoms of the meta-nitro group.

## 1. Introduction

Aminophenols are implicated in the synthesis of pharmaceutically active compounds [1]. For instance, *p*-aminophenol is an important intermediate in the preparation of several analgesic and antipyretic drugs, such as paracetamol, acetanilide, and phenacetin [2]. In addition, aminophenols are key ingredients in the production of metal-complex dyes and are used in corrosion inhibition, hair-dying agents, and polymer production [1,2]. Due to their diverse applications, the development of efficient processes for the synthesis of aminophenols has gained increasing attention.

One method of synthesizing aminophenols is via the reduction of nitrophenols. Nanostructured transition metal molybdate materials have shown promise as catalysts for this process [3,4]. In particular, nanostructured CoMoO_4_ provides a non-toxic and inexpensive candidate for this process [5]. CoMoO_4_, which may exist in several phases including a low temperature α-phase and a high temperature β-phase, [3,5,6,7] is used as a catalyst in many chemical [8] and petrochemical processes such as cracking, hydrogenation, dehydrogenation, and hydrodesulfurization (HDS) [6,9]. The catalytic properties of CoMoO_4_ depend on the structure of the material, which in turn depends on the method of preparation [10]. Various methods have been developed for the synthesis of CoMoO_4_ including precipitation, sol–gel, solid state reaction, hydrothermal, complete evaporation of a polymer-based metal-complex precursor solution, microemulsion and sonochemical [11,12,13,14,15,16].

Several materials were tested in the reduction reaction of the nitrophenol isomers. The ferrites materials CuFe_2_O_4_ and NiFe_2_O_4_ were studied by Goyal et al. and found that the copper containing ferrite was a better catalyst for these reactions [17]. Moreover, Nandanwar et al. investigated the efficiency of CuO/γAl_2_O_3_, which showed good catalytic activity [18]. Ni/C black composite material was also tested as a catalyst for this reaction by Xia et al., and low efficiency was observed [19]. Better results were found in a study conducted by Oudghiri on the iron molybdate Fe_2_(MoO_4_)_3_ [20]. Ghorai et al. worked with a mixed molybdate phosphate of cobalt, copper, and chromium that was were found to efficiently reduce 4-NP [21]. In recent years, the application of CoMoO_4_ to the catalytic degradation of organic dyes has started to receive increasing attention. For instance, CoMoO_4_ nanoparticles prepared via the microemulsion method have been shown to be quite effective at eliminating reactive black (RB) 8 dye from aqueous solution, with a decolorization efficiency of 91.47% [12].

In this study, nanostructured β-CoMoO_4_ catalysts were prepared via the thermal decomposition of an oxalate precursor prepared in the solid state using a new method. The obtained powder was found to be formed of nanoparticles. The freshly synthesized β-CoMoO_4_ nanoparticles were tested as catalysts in the reduction of three aminophenol isomers (2-NP, 3-NP, and 4-NP) to their corresponding aminophenol isomers.

## 2. Material and Methods

### 2.1. Materials

Cobalt nitrate (Co(NO_3_)_2_·6H_2_O), ammonium molybdate ((NH_4_)_6_Mo_7_O_24_·4H_2_O), and oxalic acid (H_2_C_2_O_4_·2H_2_O) were obtained from Aldrich (St. Louis, MO, USA). All the chemicals were of analytical grade and used without further purification.

### 2.2. Catalyst Preparation

The cobalt molybdate catalyst (CoMoO_4_) was prepared by thermal decomposition of an oxalate precursor in a tubular furnace (open on both sides) at 550 °C for 2 h. The oxalate precursor was obtained by the solid state reaction of a well-ground mixture of cobalt nitrate (Co(NO_3_)_2_·6H_2_O), ammonium molybdate ((NH_4_)_6_Mo_7_O_24_·4H_2_O), and oxalic acid (H_2_C_2_O_4_·2H_2_O) in the molar ratio 1:1/7:5, respectively, on a hotplate at 160 °C for 1 h [22]. During heating, an orange/brownish gas is evolved, indicating the formation of NO_2_ gas due to the reduction of the nitrate anions. In the meantime, the mixture takes a dark blue coloration confirming the reduction of the molybdenum (+VI) to lower oxidation states (+V or +IV). The oxalic acid acted not only as a reducing agent but also as a complexing agent leading to the formation of the oxalate complexes of the existing metals [23,24]. The advantage of this preparation method is that it occurs in the solid state without adding any solvent. There is no factor to control other than the temperature of the precursor formation at 160 °C and the heating temperature at 550 °C. No strict conditions such as higher temperatures or pressures are needed. Moreover, the time of the preparation of the nanoparticles is limited to a few hours with a yield of almost 100%. All synthesis methods encountered in the literature require the use of solvents for the filtration and drying stages. In the solid-state reaction between MoO_3_ and Co_3_O_4_, higher temperatures as well as regrinding mixture and annealing stages are needed to obtain improved homogeneity.

### 2.3. Characterization

In order to study the decomposition with temperature of the prepared oxalate precursor, thermal analyses were conducted on an SDT Q600 instrument (Ta Instruments, Hayesville, NC, USA). Infrared spectroscopy studies were conducted on a Shimadzu 8400S (Shimadzu, Tokyo, Japan), to confirm the oxalate complex formation. To verify the crystallinity and the purity of the prepared cobalt molybdate, the X-ray diffraction patterns were recorded on a Shimadzu X-ray diffractometer 6000 (Shimadzu, Tokyo, Japan), using Cu Kα radiation (1.5406 Ǻ) equipped with a Ni filter. The patterns were recorded in the range of 10–80° in 2θ. Crystallites size was calculated using the Scherer formula D_XRD_ = 0.9 λ/(B cosθ), where λ is the Cu Kα wavelength, B is the full width at half maximum (FWHM) expressed in radians, and θ is the Bragg angle. Particle sizes could also be estimated using the equation D_BET_ = 6000/d.S, where d is the density and S is the specific surface area calculated from the adsorption/desorption isotherms that were obtained from a Micrometrics ASAP 2020 (Micromeritics, Norcross, GA, USA), surface area and porosity analyzer. The variation of the nitrophenol isomer (2-NP, 3-NP and 4-NP) concentrations during the catalytic transformation to their corresponding aminophenol isomers was measured using a Varian Cary 100 UV-visible spectrophotometer (Varian Inc., Palo Alto, CA, USA). Transmission electron microscopy (TEM) was performed using a Tecnai-12 electron microscope (FEI, Hillsboro, OR, USA), operated at 120 kV to investigate the shape and size of the particles. For the TEM measurement, the sample was prepared by simply grinding the powder between two glass plates and bringing the fine powder into contact with a carbon-coated copper TEM grid. Energy dispersive X-ray (EDX) microanalyses were obtained using an EDAX detector coupled to a TEM instrument.

### 2.4. General Procedure for the Reduction of Nitrophenol Isomers

The prepared cobalt molybdate powder was tested as a catalyst in the reduction of three nitrophenol isomers (2-NP, 3-NP, and 4-NP) to their corresponding aminophenol isomers. In a typical test, 40 mL of an 8 × 10^−4^ M aqueous solution of sodium tetrahydroborate (NaBH_4_) were added to 40 mL of a 4 × 10^−4^ M aqueous solution of the nitrophenol isomer under continuous stirring at ambient temperature. The solution color immediately became dark yellow, with an absorption located at 415, 393, and 401 nm for the *ortho*-isomer (2-NP), the meta-isomer (3-NP), and the para-isomer (4-NP), respectively, due to the resonance stabilization of the formed nitrophenolate anions. One hundred milligrams of the cobalt molybdate catalyst was then added to the mixture under continuous stirring. The reaction was monitored via UV visible spectroscopy measurements. Absorbance intensity decreases as nitrophenolate anion concentration decreases, thus allowing us to use this relationship as a measure of the efficiency of the catalyst in this reduction reaction.

## 3. Results and Discussion

### 3.1. Complex Precursor Characterizations

**Figure 1:**
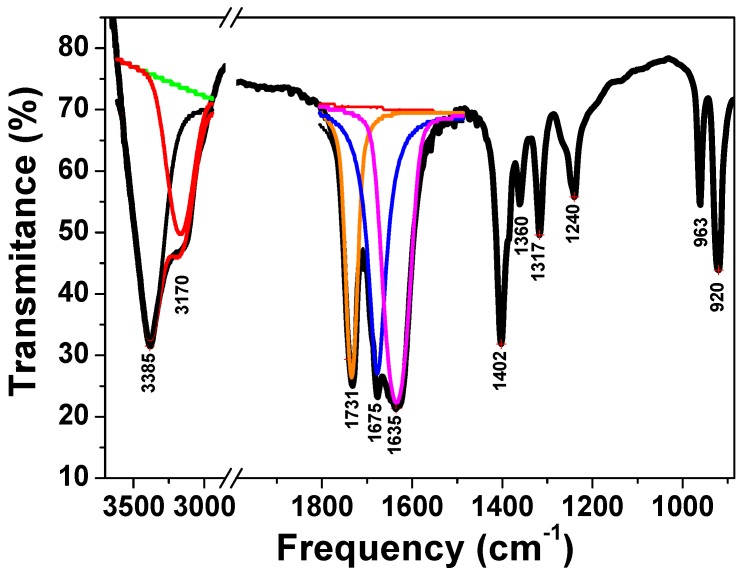
FTIR spectrum of the as-prepared oxalate precursor.

**Figure 2:**
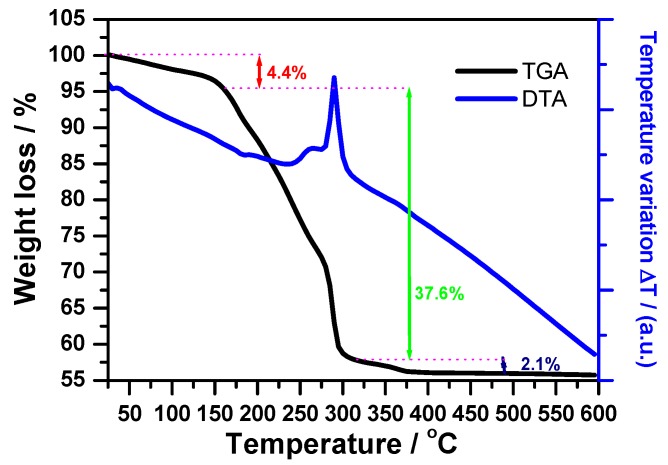
TGA and DTA thermograms recorded under air.

As the first step, the product of the solid state reaction between the mixture of Co(NO_3_)_2_·6H_2_O and the ammonium molybdate ((NH_4_)_6_Mo_7_O_24_·4H_2_O) with the oxalic acid was studied by infrared spectroscopy. The FTIR spectrum of the resulting complex is shown in Figure 1. The spectrum is similar to previous studies for oxalate complexes [25,26,27,28]. The bands at 920 and 963 cm^−1^ are assigned to the Mo=O stretching, the 1360 and 1317 cm^−1^ region can be assigned to υ(C-O) and δ(OCO), and the 1402 cm^−1^ band to the C-O stretching [28]. The spectrum also reveals the presence of a large band in the range of 1800–1550 cm^−1^. The deconvolution of this band shows bands at 1731 and 1675 cm^−1^, which can be assigned to the C=O vibration of the oxalate group [27,28] and a band at 1635 cm^−1^ corresponding to δ(H_2_O) [26]. At high frequency, the spectrum reveals a broad intense band in the 2850–3750 cm^−1^ range. The deconvolution of this broad band reveals bands at 3385 cm^−1^, which can be attributed to the OH bridging group between two metal ions [29], and at 3170 cm^−1^, which can be attributed to the stretching vibration of NH_4_^+^ ion [29,30]. Furthermore, a band at 1240 cm^−1^ can be attributed to δ(NH_4_^+^) and confirm therefore the presence of the NH_4_^+^ group in the precursor [30]. These results permit the conclusion that an oxalate complex, containing oxo molybdenum units, hydroxyl (-OH), water, and NH_4_^+^ ion, was formed. The results from the thermal analysis (TGA and DTA) studies are presented in Figure 2. The TG curve is divided in four parts with a total weight loss of 44.1% between room temperature and 380 °C. The first weight loss of 4.4% can be attributed to the crystallization water. Moreover, the second and third weight losses located between 170 and 300 °C can be attributed to the decomposition of the complex. Finally, a last small loss of 2.1% is observed between 300 and 375 °C. A similar loss in the same range was obtained in a previous study of bismuth oxalate complex containing an OH group and can be attributed to this OH group [31,32]. By compiling the results obtained by FTIR, TGA, and the possible oxidation degree of cobalt and molybdenum, a formula of the oxalate complex can be suggested as (NH_4_)CoMoO(C_2_O_4_)_2_(OH)·H_2_O. The total weight loss observed is 44.1% in comparison with the theoretical value of 45.3% for the suggested formula. This complex was subsequently thermally decomposed in air at a chosen temperature of 550 °C to obtain the resulting oxide phase.

### 3.2. Cobalt Molybdate Characterization

#### 3.2.1. X-ray Diffraction

**Figure 3:**
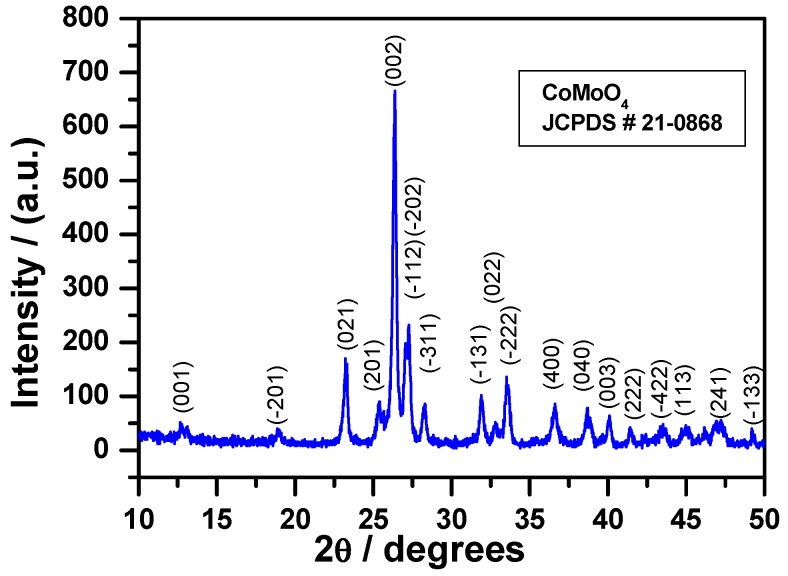
XRD pattern of the β-CoMoO_4_ nanoparticle powder obtained after calcination of the oxalate precursor at 550 °C.

The X-ray diffraction pattern of the powder prepared via this new and original method is given in Figure 3. A pure phase CoMoO_4_ was obtained. All the observed peaks could be indexed in accordance with the J.C.P.D.S. file # 21-0868. The CoMoO_4_ crystallizes in the monoclinic cell in the space group C2/m with the parameters: a = 10.21, b = 9.268, c = 7.022, and β = 106.9°. The crystallites size was calculated using the Debye–Scherer equation, assuming that the crystallites have a spherical shape. A separate diffraction peak (021) was considered for this calculation (2θ = 23.22°). The crystallites size was found to be 17.6 nm.

#### 3.2.2. Specific Surface Area Measurement

**Figure 4:**
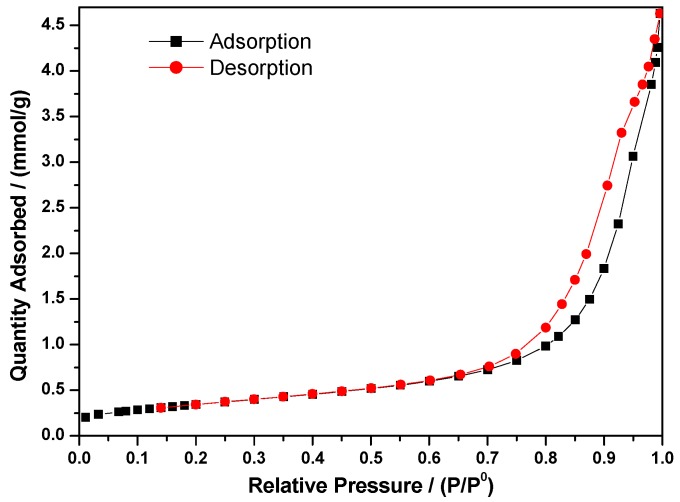
Adsorption and desorption curves obtained from the BET measurements of the β-CoMoO_4_ nanoparticles.

The synthesized cobalt molybdate (CoMoO_4_) has a BET surface of 28.35 m^2^/g, which corresponds to a particle size of 46.3 nm calculated using the equation D_BET_ = 6000/d.S, where d is the density and S is the specific surface area. It is a higher value than that calculated from the XRD pattern. On the other hand, as depicted in Figure 4, the adsorption/desorption isotherms are type IV exhibited by mesoporous solids and exhibit the characteristic H1 hysteresis loop (IUPAC classification), which is consistent with mesoporous or nanoporous materials [33,34]. The pores are cylindrical or slit shaped. The pore size was found to be of 18.8 nm, and the total pore volume found to be 0.1336 cm^3^/g using the BJH (Barrett-Joyner-Halenda) method.

#### 3.2.3. Transmission Electron Microscopy

**Figure 5:**
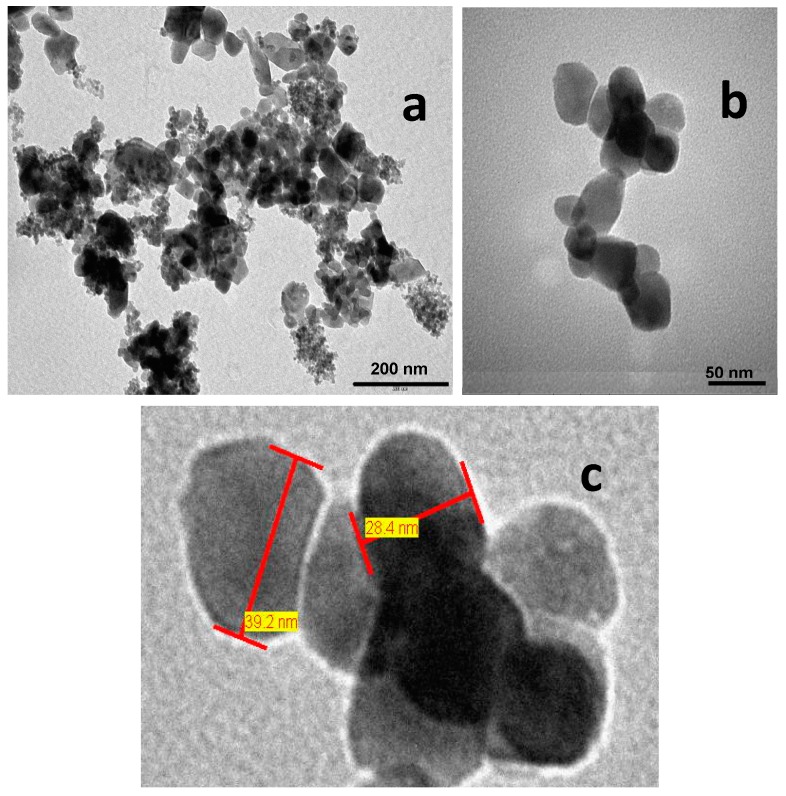
The TEM micrographs of the as-prepared β-CoMoO_4_ powder (**a**) at low magnification (×100,000), scale bar 200 nm; and (**b**) at high magnification (×500,000), scale bar 50 nm; (**c**) Measured particle size at high magnification.

Micrographs of the β-CoMoO_4_ nanoparticles were taken at low magnification showing agglomerated nanoparticles (Figure 5a). The agglomerates are about one micron in size. At higher magnification, the particle size found is in the range between 20 and 40 nm (Figure 5b,c). This value is lower than obtained from BET study (46.3 nm). This result can be explained by the fact that in BET, agglomerated particles offer less surface for the adsorption-desorption phenomena, so the calculated particle size is higher. The calculated particle size using the Scherer formula from XRD study (17.6 nm) was found lower than those found in either BET or TEM observation. This is because what was calculated was the crystallite size, which was smaller than the particle size.

**Figure 6:**
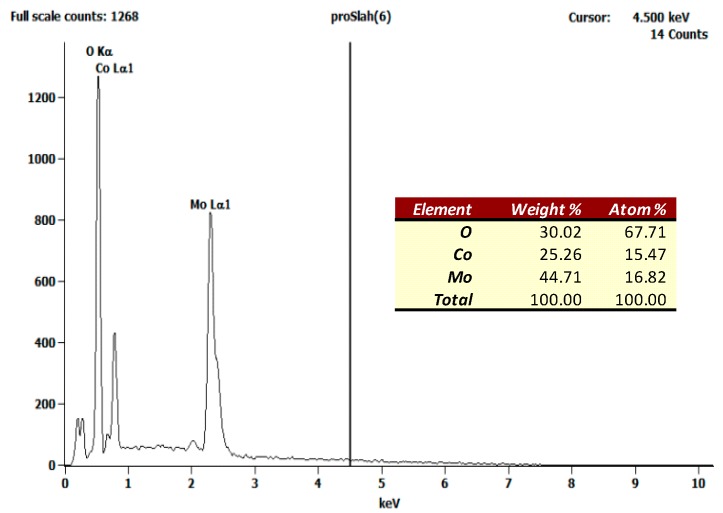
EDX spectrum of the as synthesized β-CoMoO_4_ and its atomic abundance.

In order to confirm the atomic composition and proportion in the β-CoMoO_4_ nanoparticles, a study with EDX spectroscopy was performed. Figure 6 shows the results. The abundance of oxygen, cobalt, and molybdenum were found to be of 67.71%, 15.47%, and 16.82% compared to the theoretical values 66.67, 16.67, and 16.67 respectively. 

### 3.3. Reduction of Nitrophenol Isomers

**Figure 7:**
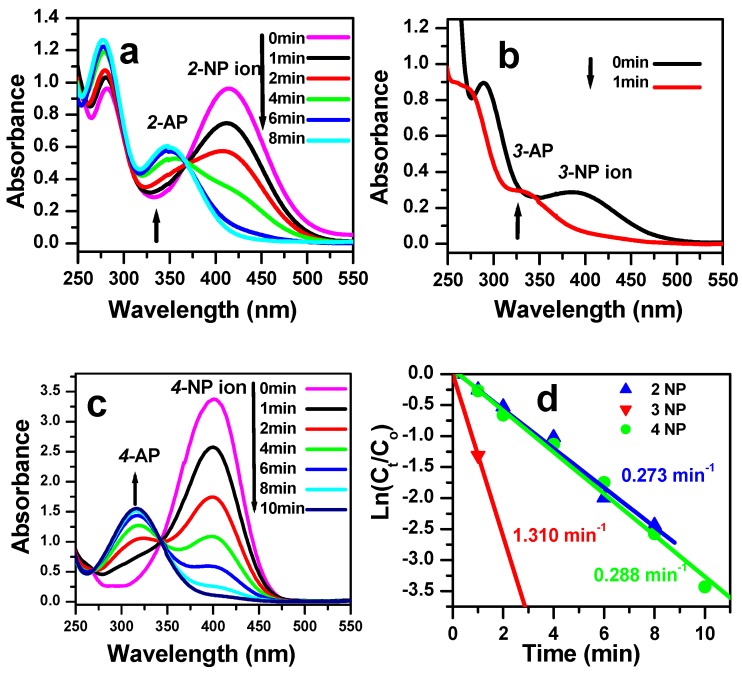
Successive UV-vis spectra of the reduction of (**a**) 2-nitrophenol solution (4 × 10^−4^ M); (**b**) 3-nitrophenol solution (4 × 10^−4^ M); (**c**) 4-nitrophenol solution (4 × 10^−4^ M) in the presence of 8 × 10^−4^ M NaBH_4_ using β-CoMoO_4_ nanocatalyst (0.1 g); (**d**) Plot of ln(C/C_0_) against reaction time for the nitrophenol isomers in the presence of β-CoMoO_4_ and determination of the reduction reaction rate constant k_app_.

The results of the catalytic reduction study are shown in Figure 7. As is clear, the efficiency of the β-CoMoO_4_ catalyst is remarkable. An instantaneous reduction happened in the case of 3-NP (Figure 7a). In contrast, the reduction reaction was slower in the case of 2-NP and 4-NP (8 min). This difference in efficiency is probably due to a higher basic character of 3-NP, where the mesomeric conjugation is more important.

An experiment was conducted to study the influence of the NaBH_4_ concentration on the rate constant of the reduction reaction. Different concentrations were taken for NaBH_4_ maintaining the same conditions as described in the protocol. When the concentration was increased to NaBH_4_/NP ratios of 50:1, 25:1, 12.5:1, and 5:1, the catalyst was decomposed by the hydride. However, when a 2:1 ratio was used, the catalyst did not react with NaBH_4_. These were the optimal conditions for this reaction.

**Figure 8:**
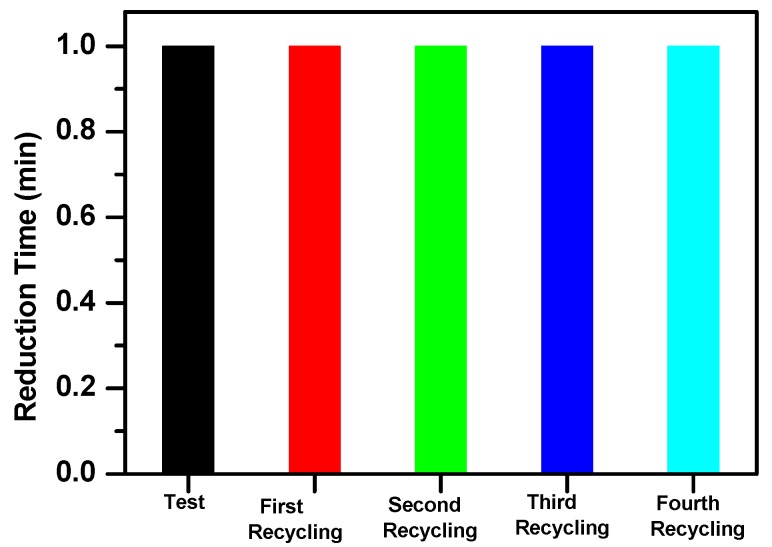
Recycled catalyst against reduction time in the reduction of 3-NP with NaBH_4_ catalyzed with β-CoMoO_4_ nanoparticles.

On the other hand, the catalyst recycling was studied for 3-NP, where a faster reduction time was observed. After the reduction test, the solution was filtered and the powder recuperated. After thorough washing with water, the powder was left to dry at 80 °C overnight and used in the next recycling test, conserving the same experimental conditions operated in the first reduction test. The recycling was repeated four times, and the results are represented in Figure 8. The reduction time of 1 min did not change in any the recycling tests, showing the good stability of the β-CoMoO_4_ nanocatalyst in this type of reduction reaction.

It is important to compare the relative reaction kinetics of the 2-NP, 3-NP, and 4-NP reduction for the catalysts synthesized in this work and those reported in the literature for other catalysts. The results are summarized in Table 1.

Importantly, this is the first time that such a high catalytic efficiency has been observed for a low-cost nanocatalyst and highly concentrated solutions of nitrophenol isomers. The observed rate constant for the simultaneous reduction of the 3-NP isomer for the β-CoMoO_4_ catalyst is relatively high and is of 1.310 min^−1^. However, definite conclusions cannot be reached from these comparisons due to the differences in the experimental conditions, such as the NaBH_4_/NP/catalyst equivalent ratio and the temperature or the reactants concentration. On the other hand, the reaction time is also a good criterion of efficiency and in our case the reaction is instantaneous. Goyal et al. have reported a similar efficiency [17] but for a lower concentration of the nitrophenol. One important point is that the reduction of the 3-NP isomer is faster than the reduction of the 2-NP isomer by approximately one order of magnitude (8/1) and the reverse situation is observed in the work of Goyal et al. This suggests that the reduction reaction mechanism is different for the β-CoMoO_4_ and CuFe_2_O_4_ catalysts.

## 4. Conclusions

β-CoMoO_4_ nanostructures with a high catalytic efficiency in the reduction of *ortho*-, *meta*-, and *para*-nitrophenol isomers to their corresponding aminophenols have been successfully prepared via the thermal decomposition of an oxalate precursor. The reduction was instantaneous in the case of 3-NP but quite a bit slower in the cases of 2-NP and 4-NP (8 min). This difference in efficiency is probably due to the higher basicity of 3-NP. A recycling test on the reduction of 3-NP showed the stability of the nanocatalyst and its high efficiency even after four cycles.

## Figures and Tables

**Table 1:** Pseudo-first order rate constants for the reduction of 2-NP, 3-NP, and 4-NP by CoMoO_4_ with other nanocatalysts reported in the literature.

Catalyst	Type	Concentration of NP (mol/L)	Reaction Time (min)	Rate Constant (min^−1^)	References
CoMoO_4_	Nanoparticles	2 × 10^−4^	8	0.273 for 2-NP	This work
			1	1.310 for 3-NP	
			8	0.288 for 4-NP	
CuFe_2_O_4_	Nanoparticles	3.6 × 10^−5^	3	3.676 for 2-NP	[17]
			1.5	0.983 for 3-NP	
			3	0.846 for 4-NP	
NiFe_2_O_4_	Nanoparticles	3.6 × 10^−5^	20	0.327 for 2-NP	[17]
			12	0.062 for 3-NP	
			16	0.118 for 4-NP	
CuO/γAl_2_O_3_	Nanocomposites	2.9 × 10^−5^	15	---- for 2-NP	[18]
			20	---- for 3-NP	
			12	0.174 for 4-NP	
Ni/C black	Nanocomposites	5.0 × 10^−4^	15	0.594 for 2-NP	[19]
			15	0.594 for 3-NP	
			15	0.5970 for 4-NP	
Fe_2_(MoO_4_)_3_	Nanoparticles	2 × 10^−4^	12	0.160 for 2-NP	[20]
			4	0.427 for 3-NP	
			9	0.323 for 4-NP	
